# Effect of Organoclay Addition on Rheological, Thermal, and Mechanical Properties of Nitrile Rubber/Phenolic Resin Blend

**DOI:** 10.3390/polym14071463

**Published:** 2022-04-03

**Authors:** Sara Shafiee, Leila Bazli, Mohammad Karrabi, Mir Hamid Reza Ghoreishy, Milad Bazli

**Affiliations:** 1 Rubber Group, Processing Department, Iran Polymer and Petrochemical Institute, Tehran 1497713115, Iran; s.shafiee@ippi.ac.ir (S.S.); leilabazli64@gmail.com (L.B.); m.karabi@ippi.ac.ir (M.K.); m.h.r.ghoreishy@ippi.ac.ir (M.H.R.G.); 2 College of Engineering, IT and Environment, Charles Darwin University, Darwin 0801, Australia; 3 School of Mechanical and Mining Engineering, The University of Queensland, Brisbane 4000, Australia

**Keywords:** nitrile butadiene rubber, phenolic resin, rheology, nanoparticles, organoclay, thermal stability, mechanical properties

## Abstract

In this study, the effects of NBR polarity and organoclay addition on the curing, rheological, mechanical, and thermal properties of an NBR/phenolic resin blend were investigated. The samples were prepared using a two-roll mill. The results showed that rheological and tensile properties improved due to the good distribution of nanoparticles, as well as the good compatibility of nitrile butadiene rubber with phenolic resin. The addition of 1.5 phr of nanoparticles to blends containing 33% and 45% acrylonitrile increased the curing torque difference by approximately 12% and 28%, respectively. In addition, the scorch time and curing time decreased in nanocomposites. Adding nanoparticles also increased the viscosity. The addition of phenolic resins and nanoparticles has a similar trend in modulus changes, and both of these factors increase the stiffness and, consequently, the elastic and viscous modulus of the specimens. Adding 1.5 phr of organoclay increased the tensile strength of the blends by around 8% and 13% in the samples with low and high content of acrylonitrile, respectively. Increasing the temperature of the tensile test led to a reduction in the tensile properties of the samples. Tensile strength, elongation at break, modulus, and hardness of the samples increased with increasing organoclay content. In addition, with increasing nanoparticle concentration, the samples underwent lower deterioration in tensile strength and Young’s modulus at different temperatures compared to the blends. In the samples containing 1.5 phr of organoclay, the thermal decomposition temperatures were enhanced by around 24 and 27 °C for low and high acrylonitrile content.

## 1. Introduction

Layered silicates or clays, among different nanofillers, occupy a leadership place in polymeric nanocomposites because of their moderate cost and commercial availability [1]. A tactoid or layered silicate consists of several single layers with regular gaps between them called galleries. These layers are held together by electrostatic forces [2,3]. Efficient polymer/clay nanocomposites are obtained when the silicate layers are separated into single layers and dispersed uniformly in the polymeric matrix. This dispersion condition, referred to as the exfoliated state, is optimal for a nanocomposite since it results in the highest improvement in physico-mechanical properties [4,5,6]. However, achieving the exfoliated morphology is arduous and the intercalated morphology is mostly obtained. In this case, the polymer chains enter the spaces between the layers (galleries), resulting in gallery expansion. To increase their interlayer spacing, a thermodynamic affinity for polymer chains and consequent enhancement of their dispersion in polymer matrices, organically modified clay, referred to as organoclay (OC), have been used. With the use of OC, both the exfoliated and intercalated morphologies are frequently achieved [7,8].

A mixture of phenolic resin and synthetic rubber has been used as a binder for friction materials of train vehicles’ frictional braking systems [9,10]. Through the use of such blends, one can create a polymeric binder having the qualities of both a hard thermosetting resin and flexible rubber. Nevertheless, broad use of this combination as a friction material binder is limited by the rubber phase’s inferior temperature stability when compared to phenolic resin [11,12,13].

Nitrile butadiene rubber (NBR) is a polar rubber that is exceptionally resistant to oil absorption because of the nitrile content. The amount of acrylonitrile in the nitrile rubber can be changed to alter its polarity and a variety of qualities. The lower the nitrile percentage, the better the low-temperature flexibility, while the higher the nitrile level, the better the resistance to aromatic hydrocarbons. Its compatibility with certain compounding ingredients can be influenced by its polarity [14]. Nevertheless, similar to other elastomers, it is not stable at high temperatures. When exposed to a high temperature and pressure flame, a strong char coating cannot be formed by the elastomer. This can be handled through the use of char-producing or flame-retardant compounds. According to Maamori et al. [15], using phenolic resin in conjunction with carbon black increased the thermal characteristics of nitrile rubber because of its high strength [16]. The polarity of the phenolic resin network results from the hydroxyl group’s existence on the benzene ring. NBR and phenolic resin are both organic compounds with polar natures that are miscible with one another. Thus, an NBR/phenolic resin blend without the incorporation of any filler improves the composite’s thermal properties and ablation resistance [15,17]. Mirabedini et al. reported that, by the addition of phenolic resin into NBR, the viscoelastic behavior of the compounds becomes more elastic and less viscous. Moreover, by increasing the phenolic resin content, crosslink density increased [17]. Nawaz et al. also demonstrated the reduced ablation rate and enhanced thermal stability with the addition of this resin to NBR [10]. Owing to the low cost of this blend, large quantities of its composite can be manufactured and it can be used in industries as a thermal insulator or flame retardant for high-temperature applications. For example, the addition of alumina nanoparticles was also reported to enhance the thermal stability of the blend [18]. Therefore, the addition of phenolic resin and nanoparticles such as OC to NBR can enhance its mechanical properties and thermal stability in order to obtain flexible materials with desirable mechanical performance.

Overall, there are limited studies considering the evaluation of the NBR rubber/phenolic resin systems, especially the impact of nanoparticle addition on the rheological, thermal, and mechanical properties of this system. The purpose of this study was the improvement of the viscoelastic and mechanical properties of this blend at high temperatures using OC nanoparticles. The influence of NBR polarity (low and high nitrile content) on the properties of the nanocomposites was also investigated. The nanoparticles were added to NBR/phenolic resin and the rheological, curing, morphological, dynamic mechanical, and thermal stability properties were investigated. The mechanical properties of the nanocomposites were also studied at 50 and 75 °C.

## 2. Materials and Methods

### 2.1. Materials and Preparation of the Samples

Table 1 lists the materials used to prepare the nanocomposites. Drying of the nanoparticles was performed in a 90 °C oven for 2 h before mixing. Commercially available NBR with two different amounts of acrylonitrile content, i.e., 33% and 45%, was used for the preparation of the samples. To improve the compounding of NBR with additives and fillers, the rubber was softened on a Polymix 200 L two-roll mill (Schwabenthan Co., Berlin, Germany) at 40 °C. The friction ratio of the two-roll mill was 1.6 and the rotation speed was 15 rpm. Then, at appropriate intervals, other components such as organo-modified montmorillonite (OMMT), activators, and antioxidants were added to the mixture and blended. At 80 °C, the powder Novolac was blended with the compound. At the end of the process, vulcanizing chemicals were introduced to the compound while it was still at 40 °C. It took 20 min to complete the full mixing process of the compound. The samples were vulcanized in a hydraulic press at a pressure of 150 kg/cm^2^ and 160 °C under controlled conditions. According to previous studies, a very small mass fraction of clay was required for the improvement of properties such as filler dispersion, the thermal degradation, stiffness, and strength of phenolic resin and NBR [19,20]. The OMMT content varied from 0 to 1.5 phr in this study. Table 2 contains information on the coding and composition of the samples.

### 2.2. XRD Analysis

The structure of nanocomposites was investigated using X-ray diffraction analysis. Nanocomposites containing OMMT nanoparticles were analyzed by a Siemens D5000 X-ray spectrometer equipped with a Cu-kα radiation source, operated at a wavelength (λ) of 1.54 A°, a current of 30 mA, and a 40 kV working voltage. The measurements were carried out at room temperature within 2θ = 0–2° with a step size of 0.02 °/s. Bragg’s equation (nλ=2dsinθ) was used to determine the distance between interplanar spacing.

### 2.3. Microstructural Study

Field emission scanning electron microscopy (FESEM) was utilized to examine the phase dispersion in NBR and the microstructure of the nanocomposites. The cross-sections of the samples that were obtained by fracturing in liquid nitrogen were examined before and after etching. By immersing the prepared samples in methanol for 72 h, the phenolic phase was extracted from the fracture surfaces. An oven was used to dry the resin-free samples before being coated with gold. Finally, the phase morphology was determined using FESEM (Tescan, MIRA3-XMU, Kohoutovice, Czech Republic) at a 30 kV voltage.

### 2.4. Rheology Measurements

To study the rheological behavior of the nanocomposites, a rubber processing analyzer (RPA 2000, Alpha Technologies Co., London, UK) was used. Prior to the curing procedure, frequency sweep tests were conducted at 50 °C, between 0.05 and 30 Hz, with a 7% strain (linear viscoelastic region). The curing test was conducted at a temperature of 160 °C, with a strain of 7% and a frequency of 2 Hz.

### 2.5. DMTA Analysis

Dynamic mechanical thermal analysis (DMTA) is used to test the mechanical properties of a polymer nanocomposite when the temperature is continuously changing. The results can be used as a technique to determine the glass transition temperature (Tg) as well as the crystallization of polymers. The dynamic–mechanical evaluation of the nanocomposites (5 cm × 1 cm × 2 mm) was performed using a Triton Tritic 2000 DMTA device from −100 to 100 °C and at a frequency of 1 Hz (as per ASTM E1640) in an air environment. The heating rate was 5 °C/min and the samples underwent three-point bending.

### 2.6. TGA Analysis

Thermogravimetric analysis (TGA) (Pyris, Perkin Elmer, Llantrisant, UK) was utilized to determine the degradation and heat stability of the nanocomposites. Approximately 5 mg of the sample was thermogravimetrically analyzed in a nitrogen environment (flow rate = 60 cm^3^ min^−1^) at temperatures ranging from 25 to 800 °C, at a heating rate of 10 °C/min.

### 2.7. Mechanical Properties

To determine the tensile properties, a tensile instrument (STM-5, Santam, Tehran, Iran) equipped with a heating chamber was employed. The experiment followed the ASTM D412 standard. The evaluation of the samples was carried out at three temperatures of 50, 25, and 75 °C, at a speed of 500 mm/min. Additionally, each sample was tested three times to determine the average values of tensile strength, modulus, as well as elongation at break.

The hardness of the samples was determined using Zwick 3100 shore hardness testers from Germany based on ASTM D-2240. The samples were approximately 4 mm thick, and the value of hardness was determined after 3 s. For each sample, three measurements were taken and the average was reported.

## 3. Results and Discussion

### 3.1. Curing Properties

**Figure 1:**
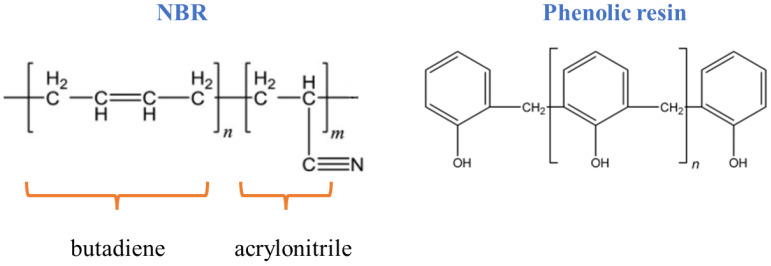
Chemical structure of NBR and phenolic resin.

Table 3 shows the curing properties that were obtained from measuring the changes in torque with time at 160 °C. As the results demonstrate, with the increase in the amount of acrylonitrile in NBR rubber, the vulcanization torque and scorch time in the rubber and blend samples increased. In the N45 sample, the percentage of butadiene groups decreased, which reduced the sites available for crosslinking. On the other hand, due to the higher polarity of the acrylonitrile groups, they formed the rigid part of the NBR rubber, resulting in an increase in the torque value. The structures of the polymers are presented in Figure 1. In the N33P and N45P samples, a reduction in the scorching time occurred because the resin helped vulcanization and thereby the formation of sulfur bridges. Because the resin contained phenol and formaldehyde groups, it helped in the formation of a complex between the activator and its vulcanizing agents and reduced the scorch time. Nevertheless, an increase in the vulcanization time in N33P and N45P compared to N33 and N45 could be associated with the resin’s interference in the reaction of sulfur bridges with each other and, therefore, the crosslink formation. Moreover, the enhancement of vulcanization torque in the blends—in particular, N45P compared to N33 and N45—could be the result of the hardening effect of the phenolic phase.

A significant increase in the torque difference was observed by adding the phenolic resin, which could be the consequence of the thermosetting behavior of the phenolic resin during the vulcanization of the rubber at high temperatures. The results of the vulcanization rate show that the rate of vulcanization is related to the percentage of acrylonitrile. There were more butadiene groups in N33 compared to N45; therefore, more active sites were provided to form sulfur bridges, crosslinks were formed in a shorter time, and the curing speed increased. However, in blends—in particular, in the N45P sample—there existed a higher interaction between the rubber chains and phenolic phase, and on the other hand, due to the increase in polarity caused by the acrylonitrile groups, the attraction among rubber chains increased and crosslinks were formed more quickly. Therefore, the rubbers showed a lower curing rate compared to the blends.

With increasing the clay nanoparticle content, the torque increased because the mobility of polymer chains and crosslinks was decreased in the presence of nanoparticles so that, by adding 1.5 phr OMMT to N33P, the enhancement of the torque difference ranged from 13.6 dN m to 15.5 dN·m. Furthermore, a reduction in the scorch and cure times was observed in nanocomposites owing to the presence of ammonium groups on the surfaces of the OMMT nanoparticles, which accelerates crosslinking [21]. Similar to the rubber and blends, N45-containing nanocomposites showed larger scorch and cure times, resulting from lower butadiene groups. In all samples, the difference in vulcanization torque was increased by the addition of nanoparticles. The amount of reinforcement and the acrylonitrile percentage in the NBR phase are the two key parameters that change the torque difference so that, by increasing the acrylonitrile percentage and the nanoparticles, the final vulcanization torque increases. The chain mobility is reduced and the torque after the completion of the vulcanization increases due to good interaction between the particles and nitrile rubber/phenolic resin blend. In all samples, the cure rate increases when increasing the content of nanoparticles. The heat transfer to the nanocomposite is improved by an increment in the density of solid nanoparticles, increasing the cure rate.

### 3.2. XRD Analysis

**Figure 2:**
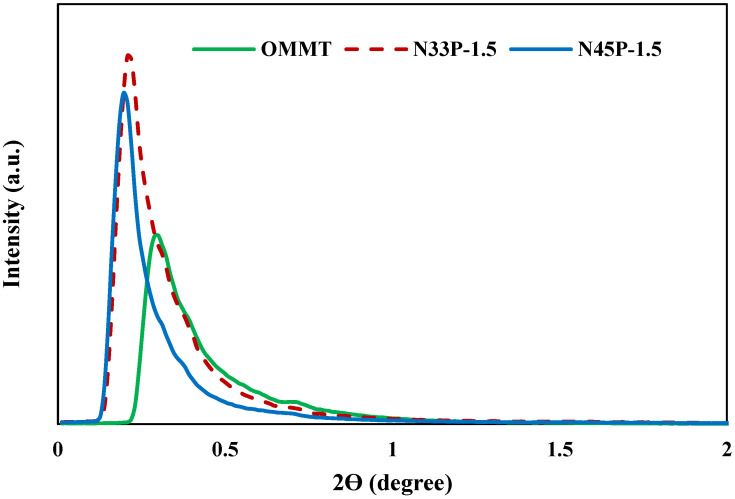
X-ray diffraction patterns of OMMT, N33P-1.5, and N45P-1.5.

**Figure 3:**
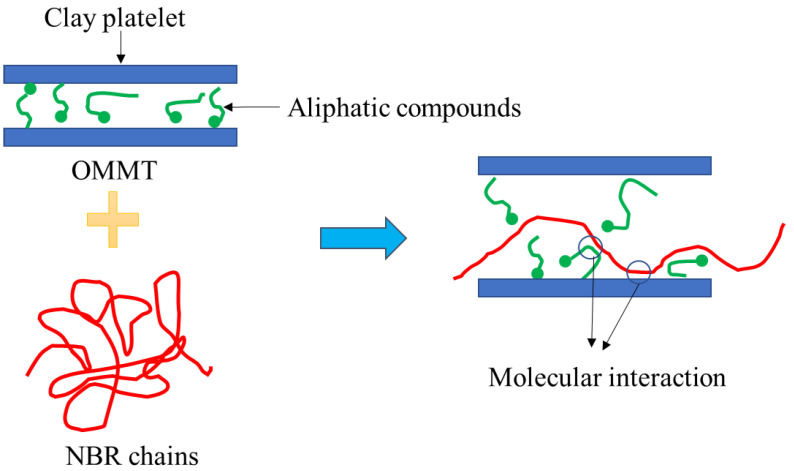
Schematic illustration of the intercalation of NBR chains in OMMT galleries.

The most important parameter that affects the rheological, mechanical, as well as physical properties of polymer-based nanocomposites is the dispersion of nanoparticles in the polymeric matrices and the ability of the polymeric chains to intercalate the galleries. Hence, in order to determine the intercalation and the increase in the galleries, tests such as XRD could be beneficial for obtaining the dispersion state and distances between layers of OMMT. In this study, Bragg’s law was used to obtain the distance between layers. The XRD patterns of OMMT and the nanocomposites containing 1.5 phr of OMMT are shown in Figure 2. The OMMT powder presents a characteristic peak in 2Ө = 0.3, which corresponds to a d-spacing of 239.7 nm. As shown in the figure, the addition of 1.5 phr of OMMT to the blends containing 33% and 45% acrylonitrile reduced the 2Ө to 0.2 and 0.19, respectively. Increasing the distance between the layers indicates the entry of polymer chains into the OMMT galleries, especially in the N45P-1.5 sample. Better interaction of the matrix containing a higher percentage of acrylonitrile, and thereby a higher degree of polarity, with the polar surface of the nanoparticles, resulted in the better dispersion of nanoparticles in the matrix. Figure 3 schematically shows the intercalation of NBR chains in OMMT galleries. Nitrile groups can interact with the polar surfaces of clay layers or polar groups of aliphatic modifiers.

### 3.3. Rheology

**Figure 4:**
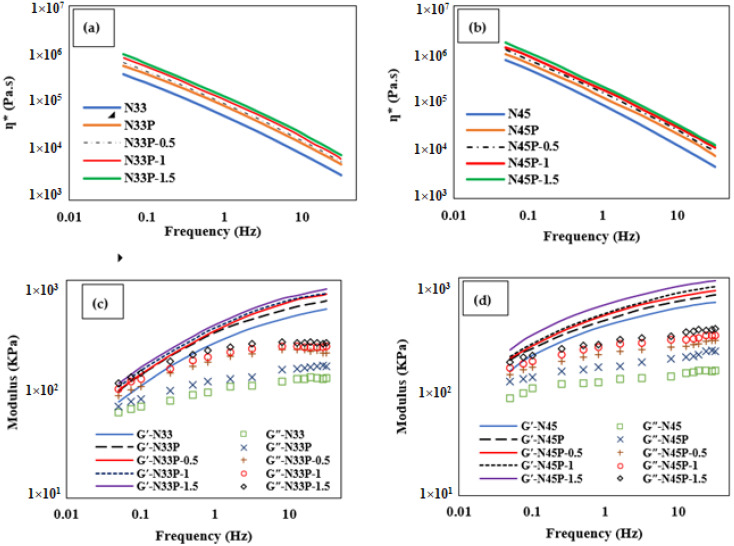
(**a**,**b**) Complex viscosity (ŋ*) vs. the frequency changes; (**c**,**d**) Variations in the storage modulus (G′) and the loss modulus (G″) with frequency at 50 °C between 0.05 and 30 Hz with 7% strain.

Figure 4a,b show the complex viscosity (ŋ*), which is obtained from real and imaginary parts, vs. the frequency changes before the vulcanization process. According to the results, the viscosity increases with the percentage of acrylonitrile. This is because the segments containing acrylonitrile groups along the rubber chains have higher stiffness. In the blends, the complex viscosities were higher than those of the rubber samples due to enhanced crosslinks in the presence of the phenolic phase. The higher density of the crosslinks increases the force required for the deformation of the blend specimens, increasing the viscosity in the frequency sweep test. At higher frequencies, the entanglements between the chains open, and the viscosity is reduced significantly as the shear rate increases simultaneously [22,23].

As shown in Figure 4a,b, ŋ* increases when increasing the content of nanoparticles because of the interaction enhancement between the OMMT nanoparticles and the matrix. At higher frequencies, the shear rate increases, leading to the disentanglement of the chains, weakening of matrix/nanoparticle interactions, and a significant decrease in the complex viscosity. In N33P and N45P samples, when the amount of OMMT nanoparticles increases to 1.5 phr, the viscosity increases by around 55% and 60%, respectively. The higher viscosity of the samples with higher acrylonitrile content results from the polar nature of OMMT nanoparticles.

Figure 4c,d depict the variations in the storage modulus (G′) and the loss modulus (G″) with frequency. As seen, both the storage and loss moduli increase with increasing frequency. The elastic behavior is higher at high frequencies. Moreover, the G′ and G″ are higher in N45P compared to N33P. Higher G″ in samples with a higher percentage of acrylonitrile can be related to the viscous movements of the chains and a decrease in the flexibility of these samples. As a result of phenolic resin addition, G′ increases due to the increase in stiffness resulting from its thermosetting behavior, and on the other hand, the reduction in its reversibility has increased the loss modulus. As the content of nanoparticles increases, the storage modulus increases, especially at lower frequencies, indicating stronger interactions between the matrix and the nanoparticles, as well as network formation and the semi-solid behavior of the nanocomposites. On the other hand, increasing G″ with the increase in the content of nanoparticles could be associated with viscous motions, particularly at higher frequencies. The viscous motions include breaking chains, weakening the matrix/nanoparticle interactions, as well as destroying the network of the nanoparticles.

### 3.4. Microstructure

**Figure 5:**
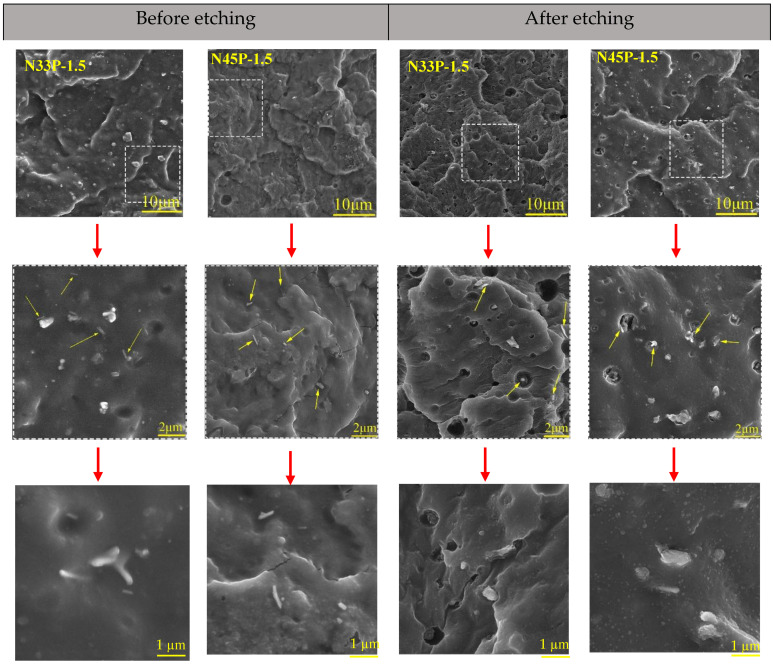
Microstructure of the blends and nanocomposites before and after etching in different magnifications.

To study the OMMT distribution, the FESEM analysis was used for the samples containing 1.5 phr nanoparticles before and after etching (Figure 5). As shown in the figure, good dispersion of the phenolic phase in the NBR phase is observed, which shows that the two polymers are highly compatible with each other. Nanocomposites that have a higher acrylonitrile percentage have more finely dispersed phase particles. In other words, higher polarity has caused a better interaction with the phenolic phase, resulting in improved compatibility. The presence of nanoparticles is shown with arrows in the figure. It is clear that OMMT nanoparticles are well dispersed in the matrix, which can lead to the production of nanocomposites with enhanced final properties. After etching, the elimination of the phenolic phase caused the formation of some pores, the size of which varied in different samples. Smaller pores are observed in N45P compared to N33P because of the better compatibility between the phenolic phase and N45 rubber. The incorporation of OMMT nanoparticles has improved the compatibility between the two phases and made the dispersed phase finer. Moreover, the tendency of clay nanoparticles to the phenolic phase (proximity of nanoclay to pores) is due to the polarity of OMMT particles.

### 3.5. Mechanical Properties

**Figure 6:**
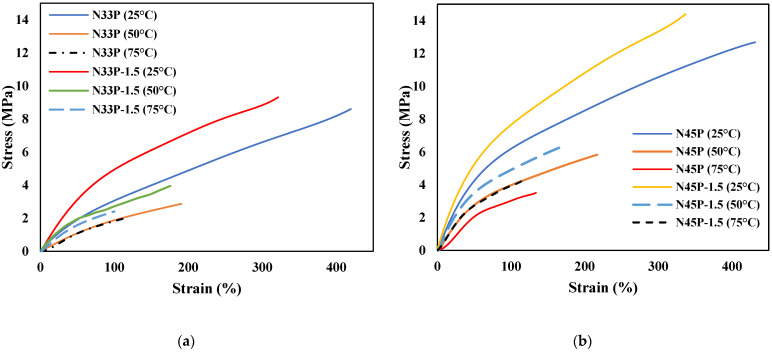
Stress–strain curves at 25, 50, and 75 °C for (**a**) N33P and N33P-1.5; (**b**) N45P and N45P-1.5.

In Table 4 and Figure 6, the results of the tensile test at 25, 50, and 75 °C are reported. As shown, the use of N45 results in better tensile properties. However, the mechanical properties of the samples are weakened significantly by increasing the temperature. Moreover, the mechanical properties change from rubber to pseudo-plastic due to the phenolic resin addition, leading to the enhancement of the tensile strength and modulus, and a reduction in elongation at break. The tensile strength of blend specimens at the three temperatures is much higher than those of rubbers. In addition, the elongation of rubber samples is higher compared to blend samples due to the greater flexibility and elasticity of rubber. Furthermore, the use of NBR with a higher percentage of acrylonitrile and the addition of phenolic resin increased the stiffness and resistance of the sample to tensile force, which led to an increase in modulus.

Various factors affect the mechanical properties of nanocomposites, including the microstructure, mixing conditions, the interaction between polymer and filler, and filler content. According to the results, the addition of OMMT improves the mechanical properties at different temperatures, while the mechanical properties are deteriorated by increasing the temperature. When the temperature of the sample increases, the distance between rubber chains increases; hence, the interaction of the chains with the nanoparticles is weakened; therefore, the stress is not transferred properly. Thus, the samples have little resistance to the applied tensile force and the nanocomposites break at lower applied forces. One of the objectives of this study was to add nanoparticles to alleviate a significant reduction in the mechanical properties with increasing temperature. Another factor that is observed is that, with increasing temperature, the decrease in tensile properties is more tangible for samples containing N45, which is due to its higher plastic properties in comparison with the samples containing N33. The difference between the glass transition temperature of the phenolic phase and the test temperature is much less than that of the NBR phase; therefore, the samples containing phenolic resin or samples containing N45 are more sensitive to the temperature change.

Increasing the concentration of nanoparticles positively affects the mechanical properties of the samples and significantly reduces the effect of the temperature increase on the tensile parameters. The improvement in mechanical properties by the addition of nanoparticles has also been reported by other researchers [24,25,26]. At a certain temperature, with increasing nanoparticle concentration, tensile properties such as strength, elongation at break, and modulus increase. However, with higher content of nanoparticles, the elongation at break, which is an indicator of the chain movement ability, is reduced due to the planar morphology of OMMT, thus leading to the restriction of the movement and elongation of the polymer chains. According to the FESM images (Figure 5), nanoparticles were relatively well dispersed in both samples containing N33 and N45, and the effective interaction of polymer chains was achieved at the interface of nanolayers. Therefore, transferring the applied stress from the matrix to the reinforcement occurs well, resulting in an increase in the tensile strength. The addition of nanoparticles to both NBR blends with different acrylonitrile percentages increases the modulus of the samples. In other words, nanoparticles with plate morphology have a need for higher stress to deform the samples, and the modulus enhances with increasing nanoparticles. Due to the higher polarity, N45-containing samples show higher interaction with the phenolic phase; thus, they have better mechanical properties.

**Figure 7:**
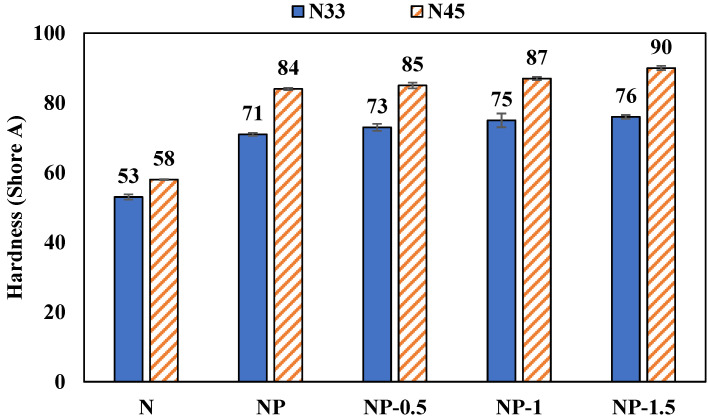
Hardness results of the samples.

The results of the hardness test are shown in Figure 7. As the figure shows, the stiffness of the rubber has been increased by the addition of phenolics to the NBR due to the increased crosslinking. On the other hand, the hardness increases with the addition of OMMT to the nitrile rubber/phenolic resin blend. Previous reports also showed the hardness increase upon increasing the proportion of nanoparticles [27]. The more difficult penetration of the test indenter into samples containing N45 is due to the higher Mooney viscosity of these samples.

### 3.6. DMTA Analysis

**Figure 8:**
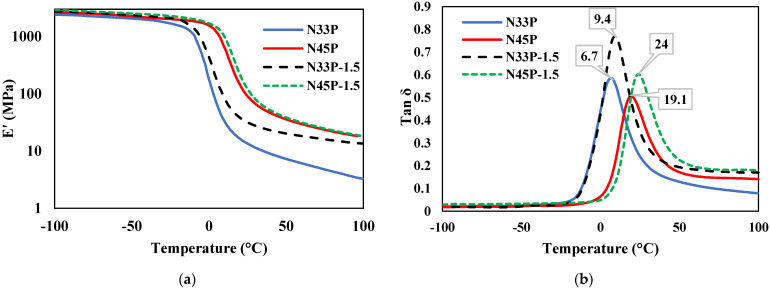
DMTA analysis results of the samples from −100 to 100 °C, at a frequency of 1, in an air environment; the heating rate was 5 °C/min; (**a**) storage modulus; (**b**) loss factor.

To evaluate the dynamic–mechanical properties of nanocomposites containing 1.5 phr OMMT, the DMTA test was used. Figure 8a shows the storage modulus variation in the temperature range of −100 to 100 °C, and the changes in tan δ (G″/G′) are shown in Figure 8b. According to the results, the blends and nanocomposites have a characteristic peak of Tg, showing the very good compatibility of these two phases, which was also observed in the FESEM images of the samples. Samples containing N45 have a Tg peak at higher temperatures, so N33P and N45P blends have Tg of 6.7 and 19.1 °C, respectively. Increasing the percentage of acrylonitrile in NBR increases the glass transition temperature. In the presence of the polar groups of acrylonitrile, the interaction among polymer chains increases; hence, a higher temperature is needed to make chains move, and therefore Tg increases. On the other hand, increasing the percentage of acrylonitrile has increased G′. The increment in hydrogen bonds in the presence of higher acrylonitrile groups and thereby higher polarity is considered the reason for the increase in G′ [28].

In the case of nanocomposites, the results show that the addition of nanoparticles to the blends increases the loss factor and shifts Tg to higher temperatures. The reason could be the increase in interfacial interactions between the matrix and the nanoparticle. The enhancement in the interactions results in the restriction of the mobility of the polymer chains and the increase in Tg [29]. The increase in the tan δ is also ascribed to the increase in filler/filler interactions [30]. Changes in storage modulus with temperature also indicate an increase with the addition of nanoparticles due to the OMMT plate morphology and their high hardness. Moreover, OMMT nanoparticles were more effective on N45-containing samples due to their polar characteristic. Adding 1.5 phr of OMMT increases the G′ of N33P and N45P by 15% and 18%, respectively.

### 3.7. TGA

**Figure 9:**
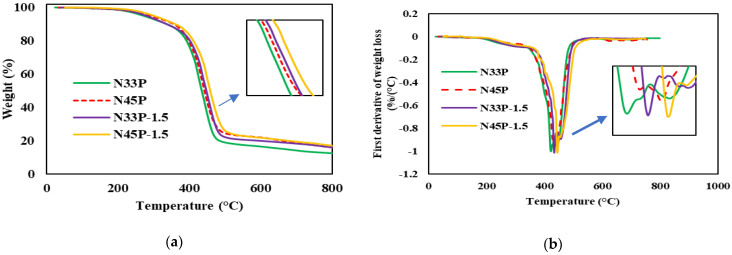
TGA analysis of the blends and nanocomposites conducted from 25 to 800 °C at a heating rate of 10 °C/min; (**a**) weight vs. temperature; (**b**) its first derivative.

TGA analysis of selected samples, its first derivative, and the decomposition temperature and char residue are given in Figure 9 and Table 5, respectively. According to the figure, all samples show thermal degradation in the temperature range of 300–400 °C because of the breakage of phenolic and NBR chains. N45P has higher thermal stability than does N33P, which is probably due to polar acrylonitrile groups, which cause higher interactions between the polymer phases, leading to their destruction at higher temperatures [31]. By adding OMMT to the blends, degradation occurs at higher temperatures so that the addition of 1.5 phr of OMMT to N33P and N45P increases the thermal degradation temperature by approximately 23.4 and 27.1 °C, respectively. Moreover, by the addition of OMMT nanoparticles, the rate of thermal decomposition is reduced. The reason is that nanoparticles play the role of a physical barrier against the diffusion of small gas molecules upon thermal degradation. When the distribution of nanoparticles is better, the thermal stability of the nanocomposite is higher. Similar results are observed in the exothermic peaks shown in the derivative curve. Additionally, in the presence of nanoparticles, the char residue of nanocomposites has increased [1,32].

## 4. Conclusions

In this study, the effects of NBR polarity and OMMT addition on the rheological properties, mechanical performance at high temperatures, and thermal stability of NBR/phenolic resin blends were investigated. The XRD patterns showed that by adding nanoparticles, the distance between the galleries increased. By adding the phenolic resin, the scorch time was reduced and vulcanization time increased. In nanocomposite samples, with an increase in the amount of OMMT nanoparticles, both the scorch and vulcanization times decreased. The final torque and the cure rate of the samples were enhanced by increasing the nanoparticles’ percentage. By the addition of phenolic resins and nanoparticles, the storage and loss modulus increased. The complex viscosity also exhibited a similar trend upon increasing the content of nanoparticles. The tensile test at ambient temperature indicated that the phenolic resin addition changed the mechanical behavior from rubber to pseudo-plastic. Moreover, the addition of nanoparticles significantly reduced the thermally induced degradation of mechanical properties. The incorporation of the nanoparticles led to an increase in the loss factor and also the glass transition temperature. Decomposition and the char residue in the TGA test increased when adding nanoparticles in N33P and N45P. This study provides an insight into the formulation and design of composite materials based on NBR/phenolic resin with high mechanical performance and thermal stability for applications in which high mechanical properties and flexibility are required.

## Figures and Tables

**Table 1:** Materials used for the preparation of the samples.

Material	Supplier	Function
NBR (N 3345 and N 4560)	Polimeri Europa, Milan, Italy	Rubber base
Phenolic resin, Novolac	Moheb Co., Qom, Iran	Plastic phase
Organoclay (OMMT); No: 682640	Sigma-Aldrich, Oakville, ON, Canada	Reinforcement
S	Rangineh Pars, Babol, Iran	Curing agent
n-cyclohexyl -2- benzothiazole sulfenamide (CBS)	Vulkacit CZ, China	Accelerator
Mercaptobenzothiazole (MBT)	Bayer, Leverkusen, Germany	Accelerator
ZnO	Rangineh Pars, Babol, Iran	Activator
Stearic acid (SA)	Unichema International, Selangor, Malaysia	Dispersion agent for ZnO
4010 NA	Bayer, Leverkusen, Germany	Antioxidant

**Table 2:** Composition and coding of the samples.

Sample	NBR33	NBR45	Novolac (phr)	OMMT (phr)	4010NA (phr)	ZnO (phr)	SA (phr)	S (phr)	CBS (phr)	MBT (phr)
N33	100	0	0	0	1.5	4	2	1.5	0.8	0.7
N45	0	100	0	0	1.5	4	2	1.5	0.8	0.7
N33P	100	0	30	0	1.5	4	2	1.5	0.8	0.7
N45P	0	100	30	0	1.5	4	2	1.5	0.8	0.7
N33P-0.5	100	0	30	0.5	1.5	4	2	1.5	0.8	0.7
N33P-1	100	0	30	1	1.5	4	2	1.5	0.8	0.7
N33P-1.5	100	0	30	1.5	1.5	4	2	1.5	0.8	0.7
N45P-0.5	0	100	30	0.5	1.5	4	2	1.5	0.8	0.7
N45P-1	0	100	30	1	1.5	4	2	1.5	0.8	0.7
N45P-1.5	0	100	30	1.5	1.5	4	2	1.5	0.8	0.7

**Table 3:** Cure parameters of the samples.

Sample	Scorch Time (min)	Cure Time (min)	$M_{H}-M_{L}\;(\mathrm{dN}\cdot m)$	Cure Rate (dN·m/min)
N33	2.7	6.5	11.9	0.9
N33P	2.4	6.8	13.6	0.9
N33P-0.5	2.3	6.8	14.0	1.0
N33P-1	2.1	6.3	14.5	1.0
N33P-1.5	1.7	5.8	15.5	1.1
N45	3.8	7.2	12.2	0.8
N45P	3.6	10.2	15.8	1.2
N45P-0.5	3.5	10.1	16.9	1.2
N45P-1	3.4	10.0	17.6	1.3
N45P-1.5	3.2	9.8	20.3	1.4

**Table 4:** Mechanical properties of the samples at 25, 50, and 75 °C.

Sample	Strength (MPa)			Elongation (%)			Modulus (MPa)		
	25 °C	50 °C	75 °C	25 °C	50 °C	75 °C	25 °C	50 °C	75 °C
N33	3.54 ± 0.32	1.61 ± 0.24	1.30 ± 0.14	627 ± 22	243 ± 12	162 ± 4	0.97 ± 0.10	0.95 ± 0.08	0.92 ± 0.04
N33P	8.60 ± 0.30	2.87 ± 0.14	1.97 ± 0.18	420 ± 8	193 ± 3	111 ± 4	2.48 ± 0.20	1.73 ± 0.11	1.47 ± 0.18
N33P-0.5	9.26 ± 0.18	3.26 ± 0.20	2.21 ± 0.20	401 ± 6	188 ± 3	114 ± 4	3.58 ± 0.25	2.13 ± 0.18	2.06 ± 0.14
N33P-1	9.64 ± 0.15	3.34 ± 0.15	2.43 ± 0.11	379 ± 10	181 ± 4	109 ± 6	4.05 ± 0.18	2.35 ± 0.14	2.21 ± 0.09
N33P-1.5	9.31 ± 0.25	3.42 ± 0.15	2.55 ± 0.12	345 ± 7	158 ± 3	87 ± 3	4.91 ± 0.36	2.38 ± 0.20	2.36 ± 0.21
N45	7.43 ± 0.61	2.26 ± 0.32	1.59 ± 0.14	773 ± 32	293 ± 18	178 ± 7	1.28 ± 0.18	2.38 ± 0.14	2.36 ± 0.09
N45P	12.68 ± 0.39	5.82 ± 0.24	3.50 ± 0.17	432 ± 4	217 ± 2	134 ± 4	5.92 ± 0.35	3.92 ± 0.15	3.27 ± 0.14
N45P-0.5	13.29 ± 0.35	6.06 ± 0.20	3.96 ± 0.15	430 ± 6	210 ± 3	149 ± 7	6.56 ± 0.25	4.31 ± 0.12	3.44 ± 0.11
N45P-1	14.07 ± 0.35	6.29 ± 0.20	4.11 ± 0.10	401 ± 8	207 ± 4	137 ± 4	6.62 ± 0.14	4.45 ± 0.16	3.75 ± 0.08
N45P-1.5	14.39 ± 0.25	6.26 ± 0.15	4.25 ± 0.20	383 ± 7	181 ± 2	125 ± 6	7.19 ± 0.28	4.66 ± 0.24	3.90 ± 0.18

**Table 5:** Thermal decomposition properties of the blends and nanoparticles.

Sample	Decomposition (Onset) Temperature (°C)	Peak Temperature (°C)	Char Residue (wt%)
N33P	373.8	422.8	12.3
N45P	380.3	431.4	15.8
N33P-1.5	397.2	434.9	16
N45P-1.5	407.4	444.7	17.1

## Data Availability

Not applicable.
